# Persistence of Anti-HB Antibodies in Healthcare Trainees: The Impact of Childhood Versus Adolescent Vaccination

**DOI:** 10.3390/vaccines13060562

**Published:** 2025-05-26

**Authors:** Luca Di Giampaolo, Luca Coppeta, Paola Borrelli, Piergiorgio Astolfi, Andrea Resta, Lucia Loffredo, Flavia Di Menno Di Bucchianico, Rocco Mangifesta, Lorenzo Ippoliti, Cristiana Ferrari

**Affiliations:** 1 Department of Innovative Technologies in Medicine & Dentistry, University “G. d’Annunzio” of Chieti-Pescara, 66100 Chieti, Italy; luca.digiampaolo@unich.it (L.D.G.); piergiorgio.astolfi@studenti.unich.it (P.A.); andrea.resta@studenti.unich.it (A.R.); lucia.loffredo@studenti.unich.it (L.L.); flavia.dimennodibucchianico@studenti.unich.it (F.D.M.D.B.); 2 Department of Biomedicine and Prevention, University of Rome Tor Vergata, 00161 Rome, Italy; luca.coppeta@uniroma2.it (L.C.); lorenzo.ippoliti@unicamillus.org (L.I.); 3 Laboratory of Biostatistics, Department of Medical, Oral and Biotechnological Sciences, University “G. d’Annunzio” of Chieti-Pescara, 66100 Chieti, Italy; paola.borrelli@unich.it; 4 Health and Safety Manager Unit, ASL02 Abruzzo, 66100 Chieti, Italy; rocco.mangifesta@asl2abruzzo.it; 5 Faculty of Medicine, Saint Camillus International University of Health Sciences, 00131 Rome, Italy; 6 PhD Program in Social, Occupational and Medico-Legal Sciences, Department of Occupational, Medicine, University of Rome Tor Vergata, Viale Oxford 81, 00133 Rome, Italy

**Keywords:** HBV, titer of anti-HB antibody, booster doses, vaccination, prevention, occupational medicine, healthcare trainees, health surveillance, healthcare workers, immunization

## Abstract

Background: Hepatitis B virus (HBV) infection remains a significant occupational health concern for healthcare workers (HCWs), including trainees exposed to biological risks. Although vaccination is the most effective preventive measure, the persistence of immunity over time and the need for booster doses remain subjects of debate. Objective: The present study aims to assess the prevalence of protective anti-HB antibody titers among healthcare trainees at the “SS Annunziata” Hospital in Chieti, comparing those vaccinated in infancy with those vaccinated during adolescence. Methods: A retrospective observational study was conducted on 2028 healthcare trainees from 2021 to 2024. Participants were divided into two groups based on vaccination timing: infancy (PED group) and adolescence (ADO group). Serological tests were performed to measure anti-HB titers, with a protective threshold set at ≥10 IU/L. Statistical analyses were conducted to evaluate differences in immunity persistence between the two groups. The results showed that the overall prevalence of protective anti-HB titers was 50.7%, with significant differences between the PED and ADO groups. Protective immunity was observed in 79.2% of individuals vaccinated during adolescence, compared to 44.6% of those vaccinated in infancy (*p* < 0.001). No significant differences in antibody persistence were found between males and females. Notably, 92.4% of participants with non-protective titers received a booster dose within two months of testing. Conclusions: The study confirms a significant decline in anti-HB titers over time among individuals vaccinated in infancy, suggesting a potential need for booster doses later in adulthood. The high adherence to vaccination recommendations among healthcare trainees is a promising finding, reinforcing the importance of continuous education and immunization programmes in healthcare settings. Further research, including longitudinal studies and additional HBV biomarkers, is necessary to optimize vaccination strategies and long-term immunity monitoring in HCWs.

## 1. Introduction

Hepatitis B virus (HBV) infection is a significant global public health problem, posing serious health risks [1]. According to the World Health Organization (WHO) Global Hepatitis Report 2024, approximately 254 million individuals are currently living with chronic HBV infection worldwide, and about 1.2 million new infections occur annually [2]. The WHO African region accounts for 63% of new hepatitis B infections, and yet only 18% of newborns in the region receive the hepatitis B vaccination recommended at birth, while the Western Pacific region accounts for 47% of hepatitis B deaths, and treatment coverage remains low [2].

HBV is transmitted through contact of mucous membranes or nonintact skin with infected blood or other body fluids (saliva, semen, and vaginal secretions) [3]. In high-endemic areas, transmission occurs mainly vertically, that is, from mother to child at birth (perinatal transmission), or during early childhood through contacts that occur between infected and uninfected children (horizontal transmission) [4].

In low-endemic areas, infection is most prevalent among adults belonging to defined risk groups including sexually promiscuous people, hemodialysis patients, drug addicts, prisoners, travellers to moderate or high endemic areas, and healthcare workers (HCWs) exposed to occupational hazards [5].

These infections are associated with severe complications, including acute flares, cirrhosis, hepatocellular carcinoma and extrahepatic manifestations, which contribute substantially to global morbidity and mortality [6]. It is estimated that cirrhosis and hepatocellular carcinoma cause 820,000 deaths per year [7].

In Europe, however, 14 million people are infected, although a trend toward reductions in acute cases has been observed in recent decades, mainly due to the implementation of mass immunization strategies [8]. In Italy, the incidence rate of acute HBV cases fell from 5 cases per 100,000 population in 1990 to 2 per 100,000 10 years later. In 2021, only 89 new cases were reported, with an incidence rate of 0.18 per 100,000 inhabitants.

It is evident that hepatitis B vaccines, which are accessible, safe and effective, represent the most efficacious means of controlling and preventing hepatitis B. From 2000 to 2019, global coverage with three doses of the vaccine increased from 30% to 85% [9]. Indeed, the effective implementation of hepatitis B vaccination programmes has resulted in a substantial decrease in HBV carrier rates and hepatitis B-associated morbidity and mortality [9]. The introduction of vaccines derived from plasma in 1982, and the subsequent advent of recombinant DNA technology in 1986, enabled the production of safer and more efficient vaccines. Since 1991, the WHO has been promoting vaccine integration into national immunization programmes, highlighting the importance of a dose administered at birth to prevent perinatal transmission [10].

In accordance with the recommendations issued by the WHO, the Italian legislative framework was amended in 1991, with the enactment of Law No. 165 [11], stipulating the mandatory vaccination against hepatitis B for all infants within the first year of life, and for adolescents within the twelfth year of age. The number of doses of the hepatitis B vaccine required to induce protective immunity varies depending on the type of vaccine and the age of the recipient. The standard three-dose hepatitis B vaccine series, which was historically administered as two initial doses given one month apart and a third dose given six months after the first, is no longer recommended. The WHO now recommends several options for vaccine schedules. The most appropriate ones for infants are as follows: one dose (monovalent) given at birth followed by two doses of vaccine at 1 and 6 months of age; at 2, 4, and 6 months of age; at 3, 5, and 11 months of age; at 8, 12, and 16 weeks and at 12 or 15 months of age; or at 6, 10, and 14 weeks of age, according to the WHO Expanded Immunization schedule [3]. Among the various globally approved vaccine schedules, the most effective ones that require minimal additional booster doses for immunization are preferred.

The prevalence of HBV infection among HCWs is up to four times higher than in the general population [10]. According to the WHO, about 5.6 percent of HCWs worldwide are infected with HBV, and 62,000 of these infections result from contact with infected blood [12]. Occupational accidents related to occupational biological exposures (OBH) pose a significant risk, with the possibility of contamination during patient care through accidental needle sticks or other contaminated injuries [5]. Prevention of HBV infections among HCWs requires standard precautionary measures in addition to vaccination [10]. Such measures include thorough hand washing, proper disposal of toxic instruments and waste, use of devices with retractable needles, needle protection systems, and use of personal protective equipment (PPE), including gloves, insulating clothing, and face protection. Despite the limited effectiveness of antiviral treatments, the preventive approach is the safest method to reduce the spread of infection [13].

After completion of vaccination, IgG antibodies to HBsAg (anti-HBs) are used as a marker of successful immunity against HBV. An anti-HB antibody concentration of 10 mIU/mL or higher, measured 1 to 3 months after administration of the last dose of the primary vaccination series, is considered a reliable indicator of protection against HBV infection [5,14]. HCWs who have completed the HBV vaccination programme and achieved an anti-HB antibody value of 10 mIU/mL or higher are considered protected against infection; if <10 mIU/mL, a booster dose (4 dose) is required. Among healthcare providers, measurement of the anti-HB assay is a recommended measure to demonstrate vaccination coverage to HBV.

The duration of protection conferred by the hepatitis B vaccine is not yet precisely defined. Among children who complete the three-dose primary vaccine series and are protected by presenting with an anti-HB titer of 10 mIU/mL or greater, 15–50% have low or undetectable antibody concentrations 5–15 years after vaccination [15].

Despite the existence of numerous studies that have demonstrated the efficacy of hepatitis B vaccination in providing long-term protection, uncertainties persist regarding the management of individuals exhibiting declining or undetectable antibody titers, particularly among HCWs who are exposed to occupational risk. As indicated by previous research, immunological memory has been demonstrated to persist even in the absence of detectable antibodies [16,17]. Nevertheless, there is ongoing debate surrounding the necessity of routine post-vaccination monitoring and booster administration [18,19,20].

The aim of this study is to evaluate the prevalence of protective antibody titers among a group of trainee residents attending internship at the “SS Annunziata” Hospital in Chieti by comparing coverage rates between those vaccinated in their first year of training and those vaccinated during adolescence. This assessment will help track the evolution of immunity coverage over time.

## 2. Materials and Methods

### 2.1. Sample

This retrospective observational study was conducted at the Department of Occupational Medicine in Chieti, Italy, in collaboration with the department of Biomedicine and Prevention of University of Rome Tor Vergata.

This study analyzed students enrolled in various Medical Faculty programmes—including Medicine and Surgery, Nursing, Physiotherapy, Dentistry, Dental Hygiene, Dental Practice Assistance, Biomedical Laboratory Techniques, Obstetrics, Dietetics, Speech Therapy, Orthoptics and Assistance in Ophthalmology, Healthcare, Cardiovascular Pathophysiology and Vascular Perfusion Techniques, Occupational Therapy, and TSRM—as well as Medical and Surgical Area Residents. All participants were exposed to biohazards during their internship period.

Between 2021 and 2024, a total of 2028 students and residents were enrolled during the mandatory health surveillance period under Italian Legislative Decree 81/08, as amended. The University Health Protocol includes a preventive medical examination and first-level blood tests, including a complete blood count (CBC) with leukocyte formula, liver and kidney function tests, blood glucose levels, and serological tests for HBV, HCV, and HIV, as well as the Mantoux test and any additional tests for specific risks. The measurement of anti-HB titers was conducted using the Alinity Anti-HBs Reagent Kit—Abbott (ELISA assay) in accordance with the manufacturer’s instructions. The tests were conducted at the analytical laboratory of “P.O. SS Annunziata di Chieti”. Informed consent for personal data processing and a privacy policy statement were provided to all participants.

The vaccination schedule, in accordance with national guidelines, consists of administering three doses at pediatric age (0–1–6 months). For individuals not vaccinated at a pediatric age, the three doses are administered during adolescence. Italian Legislative Decree 165/1991 [11] introduced mandatory hepatitis B vaccination for newborns.

Participants were divided into two groups: the first group included subjects vaccinated in their first year of life (PED group), while the second group included those vaccinated during their 12th year of life (ADO group).

Age was calculated as the difference between the year of birth and the year of the survey (2024). As such, it was used to describe the characteristics of the two groups studied, in particular the time elapsed between vaccination and the time of the survey.

An anti-HB titer ≥ 10 IU/L was considered protective for all subjects, based on CDC and ACIP recommendations [21], while an anti-HB titer < 10 IU/L was deemed non-protective.

### 2.2. Statistical Analysis

Descriptive analyses were conducted using mean and standard deviation (±SD) or median and interquartile range (IQR) for quantitative variables, while frequencies and percentages were used for qualitative variables. The normality of data distribution was assessed using the Shapiro–Wilk test. Associations between categorical variables were examined using Pearson’s χ^2^ test or Fisher’s exact test, while Student’s *t*-test for paired data was applied to continuous variables. Statistical significance was set at α ≤ 0.05.

All analyses were performed using Stata software v18 (StataCorp, College Station, TX, USA).

## 3. Results

Of the 2028 trainees included in the study, 63.9% were female, with a mean age of 25.89 ± 6.10 years. A total of 1754 (86.5%) had received HBV vaccination in the neonatal period (neonatal), while 274 (13.5%) had been vaccinated at the age of 12 years (childhood), and 49.3% with protective titer (≥10 IU/L). The characteristics of this population are showed in Table 1.

A comparison between the two periods (ADO vs. PED) revealed significant differences in mean age (*p* < 0.001) and vaccination coverage (*p* < 0.001) (Table 2).

We did not find a different distribution of coverage between males and females (*p* = 0.183).

In the ADO group, the vaccination coverage rate was 78.85% in males, while in females it was 79.41%, showing independence between gender and antibody protection rate (*p* = 0.911) while in the PED group, the anti-HbsAg protection rate is 46.66% for males and 43.43% for females, showing independence between gender and protection rate in the POST-1992 group (*p* = 0.192) (Table 3).

All those identified as being unprotected against HBV were offered a booster dose of the HBV vaccine as part of a wider effort to increase immunity levels and prevent potential outbreaks. Of these 1029 individuals, a significant proportion, 92.4%, received the booster dose within two months of the antibody titer measurement. Preliminary data from a subgroup of participants indicated a seroconversion rate above 95% after the administration of booster doses, demonstrating the presence of effective immune memory and an anamnestic response among healthcare trainees.

## 4. Discussion

In our study, 20.8% of students who were vaccinated at age 12 (ADO) and 55.4% of students vaccinated in the neonatal period (PED) show a non-protective titer of anti HBs. The difference is statistically significant (*p* < 0.001). As suggested by previous studies, this finding may be attributable to a more robust immune system response in adolescents than in infants. This is probably related to the maturation of the immune system in the ADO [2,3,22]. This finding may be influenced by the time elapsed between the last vaccination and the antibody titer assessment, which differs between the two groups. Several studies emphasize that, regardless of these differences, vaccination in the first year of life remains essential to ensuring early protection in childhood [23,24].

The study also shows that the duration of vaccination coverage is not affected by gender. This stratification between males and females further underscores the independence between gender and antibody titer also emphasized by other studies [25,26]. In particular, in a sample similar to the sample examined in our study, measles immunity was also found to be completely independent of gender [27].

Evaluation of the duration of immunity leads to results that are still unclear. Some researchers have reported a progressive decline with age in the anti-HB protective titer emphasizing the need for booster with a booster dose, to be administered in the morning as it would stimulate a better immune response in susceptible individuals than in those who receive booster dose administration in the afternoon [28,29]. However, in agreement with other studies, after an initial response to the vaccine, immunological memory would still ensure an adequate level of protection despite the antibody titer being non-protective (antiHBs < 10 IU/L). By virtue of this, a booster would not be necessary [20,22,30].

The findings of this study must be interpreted in light of several considerations. Despite the fact that individuals with anti-HB titers of less than 10 IU/L are not considered to be protected according to conventional criteria, it is crucial to acknowledge the persistence of immunological memory. This memory can subsequently trigger an effective immune response upon exposure to HBV. Consequently, low or undetectable antibody levels do not necessarily indicate an absence of protection. The WHO and CDC currently advise against the administration of routine hepatitis B booster doses to immunocompetent individuals [31,32]. Nonetheless, within high-risk occupational contexts, such as healthcare environments, the monitoring of anti-HB titers and the provision of booster doses can be regarded as a precautionary strategy. This approach is intended to ensure comprehensive protection and to maintain accurate documentation of immunity.

This observation highlights the issue of non-compliance with vaccination programmes, a phenomenon that has been observed among healthcare workers, often attributable to concerns regarding potential adverse effects and uncertainty surrounding the programmes’ effectiveness [33].

Specifically, we considered responses to vaccine programmes against exanthematous diseases, which, like hepatitis B vaccination, are administered during childhood. Indeed, we can refer to previous studies on samples of HCWs that have similar characteristics compared to the sample examined in this study.

In the samples examined, antibody coverage against measles, given by MPR vaccination administered in childhood, decreases over the years, thus increasing its potential risk of hospital transmission [12]. In addition, the lack of a protective IgG antibody titer is more evident in senior students [27]. Although coverage levels in Italy are still suboptimal, an increase in measles coverage from 78.7% in 2018 to 93.72% in 2022 is detectable due to the increased focus of national mandatory vaccination policies introduced in the post-Covid era [34].

For mumps, the percentage of unprotected HCWs would be particularly high while that of immune individuals would be well below the WHO threshold established for herd immunity (95%) [27].

The health professions trainees participating in this study were informed of the importance of vaccination with booster doses. This initiative was particularly important given the critical role of vaccination in controlling HBV transmission, especially in at-risk populations or in healthcare settings where exposure is more likely. Of these 1029 individuals, a significant proportion, 92.4%, accepted the offer to receive the booster dose within two months of the antibody titer measurement. This high acceptance rate reflects both the effectiveness of the communication strategies used to inform individuals about the importance of HBV immunity and, perhaps, an underlying awareness among the target population of the risks associated with HBV infection. The uptake of the booster vaccine can be seen as a positive outcome in terms of public health compliance and vaccine confidence, suggesting a generally favourable attitude towards preventive health measures when clear guidance and access are provided. It also underscores the value of targeted immunization campaigns in reducing immunity gaps and reinforces the critical role of immunization programmes in disease prevention efforts.

The result of our study data emphasises the importance of providing accurate information and ongoing training for healthcare workers in order to address concerns and improve adherence to vaccination programmes. Educating workers about the safety and effectiveness of vaccines is crucial in ensuring better participation and ultimately protecting both healthcare workers and patients.

The study provides valuable insights into HBV immunity among healthcare trainees; however, it is important to address the limitations of the study in future research. Firstly, the sample diversity is limited because the participants are drawn from students and resident doctors from both medical and surgical fields, which, while ensuring easy accessibility, may result in a lack of diversity among the subjects. To improve the generalizability of the findings, it would be beneficial to expand the study to include professionals from different regions and healthcare institutions. Another limitation is the exclusive reliance on anti-HB titers to assess immunity, without considering other HBV markers such as HBsAg or anti-HBc. Including these markers would allow for a more comprehensive evaluation, distinguishing between vaccine-induced immunity, natural immunity, and potential occult infections. Another potential limitation is the time-lag bias, since individuals vaccinated in infancy had a longer interval since their last dose compared to those vaccinated during adolescence. This finding offers a potential explanation for the observed variations in anti-HB persistence and should be taken into account when interpreting the results.

A further limitation of the study is that confounding factors such as body mass index (BMI), smoking status, and comorbidities were not collected. It is recommended that future research incorporate these variables in order to facilitate a more comprehensive understanding of their potential influence on vaccine-induced immunity. In addition, further studies are also needed to follow antibody persistence and response to booster doses over several years, which would provide more robust conclusions.

## 5. Conclusions

This study highlights the importance of monitoring HBV immunity in healthcare trainees, a group at an increased risk of occupational exposure to the virus. The results show a significant difference in antibody persistence between those vaccinated in infancy and those vaccinated in adolescence, highlighting the potential need for booster doses in certain cases. By shedding light on the long-term effectiveness of the HBV vaccination programmes, this research provides valuable data to inform public health policy and occupational health strategies. Ensuring adequate immunity among healthcare workers is essential to minimise the risk of HBV transmission in clinical settings, thereby protecting both healthcare workers and patients.

An encouraging aspect of this study is the high uptake of the vaccine among healthcare workers, with 92.4% of those who needed a booster dose receiving it within two months. This demonstrates a high level of compliance with vaccination programmes in this population, probably due to greater awareness of occupational risks and the importance of vaccination in healthcare settings. Promoting vaccine education and reinforcing the role of immunization in preventing HBV transmission will be critical to maintaining high coverage rates and improving compliance in the wider healthcare workforce.

## Figures and Tables

**Table 1:** Population’s characteristics.

Gender, n (%)	
*F*	1296 (63.9%)
*M*	732 (36.1%)
Age, years	25.89 ± 6.10
Vaccination period, n (%)	
Pediatric age (PED)	1754 (86.5%)
Adolescence (ADO)	274 (13.5%)
HBsAb, n (%)	
Non-Protective titer (<10 IU/L)	1029 (50.7%)
Protective titer (≥10 IU/L)	999 (49.3%)

Data expressed as mean ± SD or n (%).

**Table 2:** Stratified sample characteristics by period (*n* = 2028).

	ADO (*n* = 274)	PED (*n* = 1754)	*p*-Value *
Gender, n (%)			
F	170 (62.0%)	1126 (62.2%)	0.490
M	104 (38.0%)	628 (35.8%)	
Age, years	37.85 ± 5.15	24.03 ± 3.63	<0.001
HbsAb, n (%)			
Non-Protective titer (<10 IU/L)	57 (20.8%)	972 (55.4%)	<0.001
Protective titer (≥10 IU/L)	217 (79.2%)	782 (44.6%)	

Data expressed as mean ± SD or n (%); * *p*-value calculated by Pearson’s chi-square test or Student’s *t*-test.

**Table 3:** Sample characteristics stratified by gender within each period (*n* = 2028).

	ADO (*n* = 274)			PED (*n* = 1754)		
	Females (*n* = 170)	Males (*n* = 104)	*p*-Value	Females (*n* = 1126)	Males (*n* = 628)	*p*-Value *
Age, years	38.20 ± 4.91	37.27 ± 4.49	0.151	23.87 ± 3.67	24.31 ± 3.53	0.013
HbsAb, n (%)						
Non-Protective titer (<10 IU/L)	35 (20.59%)	22 (21.15%)	0.911	637 (56.57%)	335 (53.34%)	0.192
Protective titer (≥10 IU/L)	135 (79.41%)	82 (78.85%)		489 (43.43%)	293 (46.66%)	

Data expressed as mean ± SD or n (%); * *p*-value calculated by Pearson’s chi-square test or Student’s *t*-test.

## Data Availability

The data supporting the reported results can be made available upon request to the authors.

## References

[1] Morais L.Q., Motta-Castro A.R.C., Frota O.P., Contrera L., Carvalho P.R.T., Fernandes F.R.P. (2016). Hepatite B em profissionais de enfermagem: Prevalência e fatores ocupacionais de risco. Rev. Enferm. UERJ.

[2] World Health Organization (2024). Global Hepatitis Report 2024: Action for Access in Low- and Middle-Income Countries: Web Annex: Method for Global Reporting on Disease Burden and Service Coverage Data for Viral Hepatitis B and C.

[3] Van Damme P., Ward J.W., Shouval D., Zanetti A., Plotkin S.A., Orenstein W., Offit P.A., Edwards K.M. (2017). Hepatiti B Vaccines. Plotkin’s Vaccines.

[4] Chang M.H. (2007). Hepatitis B virus infection. Semin. Fetal Neonatal Med..

[5] Schillie S., Vellozzi C., Reingold A., Harris A., Haber P., Ward J.W., Nelson N.P. (2018). Prevention of hepatitis B virus infection in the United States: Recommendations of the Advisory Committee on Immunization Practices. MMWR. Recomm. Rep..

[6] Hsu Y.C., Huang D.Q., Nguyen M.H. (2023). Global burden of hepatitis B virus: Current status, missed opportunities and a call for action. Nat. Rev. Gastroenterol. Hepatol..

[7] World Health Organization (2019). Hepatitis B vaccines: WHO position paper, July 2017—Recommendations. Vaccine.

[8] Stroffolini T., Morisco F., Ferrigno L., Pontillo G., Iantosca G., Cossiga V., Crateri S., Tosti M.E., The Seieva Collaborating Group (2022). Effectiveness of Hepatitis B Vaccination Campaign in Italy: Towards the Control of HBV Infection for the First Time in a European country. Viruses.

[9] Hepatitis B., Pattyn V.J., Hendrickx G., Vorsters A., Van Damme P. (2021). Centre for the Evaluation of Vaccination. J. Infect. Dis..

[10] Auta A., Adewuyi E.O., Kureh G.T., Onoviran N., Adeloye D. (2018). Hepatitis B vaccination coverage among health-care workers in Africa: A systematic review and meta-analysis. Vaccine.

[11] Ministerial Decree 165 of 04/10/1991. https://www.who.int/publications/i/item/9789240091672.

[12] Prüss-Üstün A., Rapiti E., Hutin Y. (2005). Estimation of the global burden of disease attributable to contaminated sharps injuries among health-care workers. Am. J. Ind. Med..

[13] La Rotta E.I.G., Garcia C.S., Pertuz C.M., Miquilin I.O.C., Camisao A.R., Trevisan D.D., Aoki F.H., Correa-Filho H.R. (2020). Conhecimento e adesão como fatores associados a acidentes com agulhas contaminadas com material biológico: Brasil e Colômbia. Cienc. Saude Colet..

[14] Coppeta L., Pompei A., Balbi O., Zordo L.M., Mormone F., Policardo S., Lieto P., Pietroiusti A., Magrini A. (2019). Persistence of Immunity for Hepatitis B Virus among Heathcare Workers and Italian Medical Students 20 Years after Vaccination. Int. J. Environ. Res. Public Health.

[15] Jack A.D., Hall A.J., Maine N., Mendy M., Whittle H.C. (1999). What level of hepatitis B antibody is protective?. J. Infect. Dis..

[16] Propst T., Propst A., Lhotta K., Vogel W., König P. (1998). Reinforced intradermal hepatitis B vaccination in hemodialysis patients is superior in antibody response to intramuscular or subcutaneous vaccination. Am. J. Kidney Dis..

[17] Wiström J., Ahlm C., Lundberg S., Settergren B., Tärnvik A. (1999). Booster vaccination with recombinant hepatitis B vaccine four years after priming with one single dose. Vaccine.

[18] Bruce M.G., Bruden D., Hurlburt D., Zanis C., Thompson G., Rea L., Toomey M., Townshend-Bulson L., Rudolph K., Bulkow L. (2016). Antibody Levels and Protection After Hepatitis B Vaccine: Results of a 30-Year Follow-up Study and Response to a Booster Dose. J. Infect. Dis..

[19] Simons B.C., Spradling P.R., Bruden D.J., Zanis C., Case S., Choromanski T.L., Apodaca M., Brogdon H.D., Dwyer G., Snowball M. (2016). A Longitudinal Hepatitis B Vaccine Cohort Demonstrates Long-lasting Hepatitis B Virus (HBV) Cellular Immunity Despite Loss of Antibody Against HBV Surface Antigen. J. Infect. Dis..

[20] Chang Y.C., Wang J.H., Chen Y.S., Lin J.S., Cheng C.F., Chu C.H. (2014). Hepatitis B virus vaccination booster does not provide additional protection in adolescents: A cross-sectional school-based study. BMC Public Health.

[21] Advisory Committee on Immunization Practices, Centers for Disease Control and Prevention (CDC) (2011). Immunization of health-care personnel: Recommendations of the Advisory Committee on Immunization Practices (ACIP). MMWR Recomm. Rep..

[22] Trabucco Aurilio M., Mennini F.S., Ferrari C., Somma G., Di Giampaolo L., Bolcato M., De-Giorgio F., Muscatello R., Magrini A., Coppeta L. (2022). Main Predictors of COVID-19 Vaccination Uptake among Italian Healthcare Workers in Relation to Variable Degrees of Hesitancy: Result from a cross-sectional online survey. Trop. Med. Infect. Dis..

[23] Baghianimoghadam M.H., Shadkam M.N., Hadinedoushan H. (2011). Immunity to hepatitis B vaccine among health care workers. Vaccine.

[24] Osiowy C. (2018). From infancy and beyond… ensuring a lifetime of hepatitis B virus (HBV) vaccine-induced immunity. Hum. Vaccines Immunother..

[25] Di Giampaolo L., Costantini E., Di Nicola M., Porreca A., D’amore G., Coppeta L., Mangifesta R. (2022). Titer of anti-HBs in health professions trainees: Prevalence of antibody coverage in a University of Central Italy. Hum. Vaccines Immunother..

[26] Aurilio M.T., Iannuzzi I., Di Giampaolo L., Pietroiusti A., Ferrari C., Coppeta L. (2020). Cross-sectional Serological Study for Measles among Italian Medical Students in 2020. Open Public Health J..

[27] Floreani A., Baldo V., Cristofoletti M., Renzulli G., Valeri A., Zanetti C., Trivello R. (2004). Long-term persistence of anti-HBs after vaccination against HBV: An 18 year experience in health care workers. Vaccine.

[28] Coppeta L., Ferrari C., Verno G., Somma G., Trabucco Aurilio M., Di Giampaolo L., Treglia M., Magrini A., Pietroiusti A., Rizza S. (2023). Protective Anti-HBs Antibodies and Response to a Booster Dose in Medical Students Vaccinated at Childhood. Vaccines.

[29] Saffar M.J., Rezai M.S. (2004). Long-term antibody response and immunologic memory in children immunized with hepatitis B vaccine at birth. Indian Pediatr..

[30] Van Herck K., Van Damme P. The Immunological Basis for Immunization Series—Module 22: Hepatitis B. https://apps.who.int/iris/bitstream/handle/10665/77755/9789241504751_eng.pdf;jsessionid=72E624435FD359D0654C8F6F6706E2AC?sequence=1.18.

[31] Centers for Disease Control and Prevention (1990). Protection Against Viral Hepatitis Recommendations of the Immunization Practices Advisory Committee (ACIP).

[32] Poorolajal J., Hooshmand E. (2016). Booster dose vaccination for preventing hepatitis B. Cochrane Database Syst. Rev..

[33] Ferrari C., Somma G., Olesen O., Buonomo E., Pasanisi Zingarello M., Mazza A., Rizza S., Di Giampaolo L., Magrini A., Ponzani F. (2023). Measles vaccine uptake among Italian medical students compared to the pre-COVID-19 era. Hum. Vaccines Immunother..

[34] Signorelli C., Oleari F., Greco D., Ruocco G., Guerra R., Rezza G., Vaia F., Campitiello M.R., Fara G.M. (2024). The Italian National Vaccine Prevention Plans, 1999/2020–2023/2025: Challenges and obstacles to vaccine coverage goals. Comment. Ann. Ist. Super. Sanita.

